# A Case of E-cigarette or Vaping Product Use-Associated Lung Injury Mimicking Miliary Tuberculosis

**DOI:** 10.7759/cureus.22406

**Published:** 2022-02-20

**Authors:** William Lim, Maham Suhail, Keith Diaz

**Affiliations:** 1 Internal Medicine, Richmond University Medical Center, New York, USA; 2 Pulmonary and Critical Care, Richmond University Medical Center, New York, USA

**Keywords:** electronic cigarette associated lung injury, lung-injury, tuberculosis, effects of vaping, e-cigarette smoking

## Abstract

E-cigarette usage or vaping is becoming more popular as an alternative option to cigarette smoking. Vaping is associated with a wide degree of pulmonary injuries such as asthma, chronic obstructive pulmonary disease or E-cigarette or vaping product use-associated lung injury (EVALI). E-cigarette or vaping product use-associated lung injury is an acute or subacute respiratory illness that can be severe and life-threatening. Miliary tuberculosis, on the other hand, is a rare form of tuberculosis that results from hematogenous dissemination of *Mycobacterium tuberculosis, *affecting multiple organs and systems. It is characterized by the presence of small, firm white nodules resembling millet seeds. We report a case of a young patient presenting to the hospital with features suggestive of miliary tuberculosis in the CT scan of the chest. Diagnosis of EVALI was reached after extensive diagnostic workup including tuberculosis revealed negative.

## Introduction

Vaping is the process of inhaling an aerosol that is created from e-cigarettes [1,2]. With the increased use of e-cigarettes among young adults and teenagers, physicians must be familiar with its associated adverse effects. There are many adverse health effects associated with the use of vaping such as cardiovascular diseases, angina, stroke and pulmonary diseases. E-cigarette or vaping product use-associated lung injury (EVALI) is an acute or subacute respiratory illness that can be severe and life-threatening. It was initially described in 2019, and more than 2000 cases have been reported since then [3,4]. 

## Case presentation

**Figure 1:**
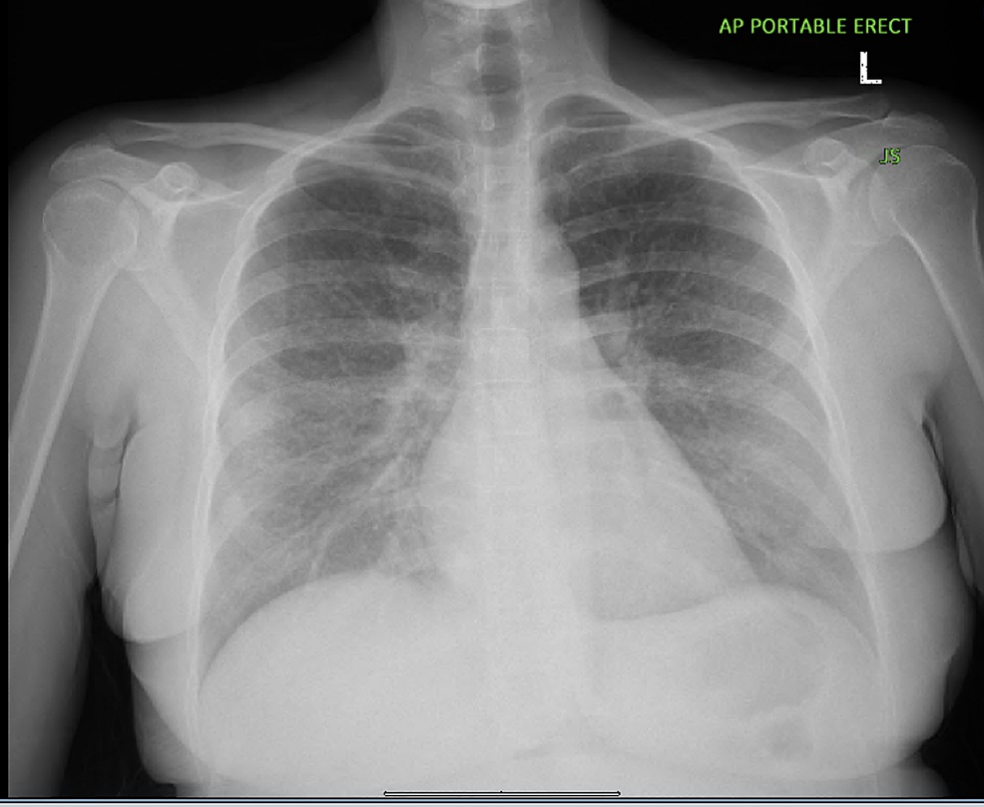
Chest X-Ray showing an ill-defined, generalized, hazy reticulonodular pattern of the lungs

A 49-year-old female with a past medical history of fibromyalgia, anxiety, seizure disorder and asthma presented to the emergency department (ED) with shortness of breath which started one week before the presentation. The patient also reported a 15-day history of fever with maximal temperature (Tmax) of 103° F, chills, and intermittent productive cough with clear phlegm associated with severe weakness, body aches and pains. The patient was a previous cigarette smoker for seven years who had switched to e-cigarettes 10 years ago. She denied alcohol drinking or recreational drug use. The patient's vital signs were as follows: temperature 98.6° F; blood pressure 151/95 mmHg; heart rate 107/minute; and oxygen saturation 95% on room air. White blood cell count was 9.8 K/uL with a neutrophilic predominance of 90% with no lymphocytosis. Basal metabolic panel (BMP) was essentially normal with a creatinine of 0.8 mg/dl and a calcium level of 8.8 mg/dl. Chest X-Ray (Figure 1) showed ​​an ill-defined, generalized, hazy reticulonodular pattern of the lungs.

**Figure 2:**
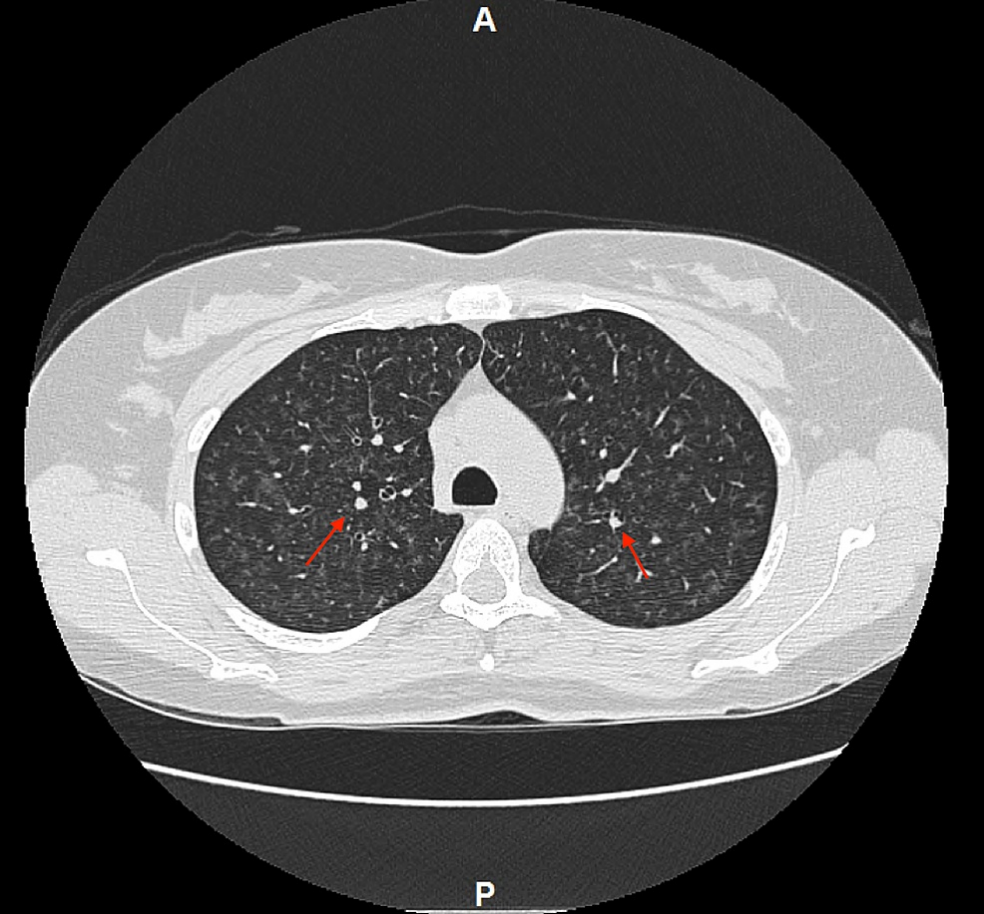
Chest CT showing innumerable pulmonary micronodules in the lungs bilaterally most prominent in the mid and upper lung zones with patchy ground-glass opacity and tree-in-bud opacities

The CT scan of the chest with contrast (Figure 2) showed innumerable pulmonary micronodules in the lungs bilaterally most prominent in the mid and upper lung zones with patchy ground-glass opacity and tree-in-bud opacities with mediastinal and bilateral hilar lymphadenopathy. Respiratory viral panels such as adenovirus, coronaviruses including coronavirus disease 2019 (COVID-19), human metapneumovirus, influenza, parainfluenza, respiratory syncytial virus (RSV) and entero/rhinovirus revealed a negative result. Blood culture and sputum culture obtained showed no growth of organisms. Ceftriaxone 1 gm intravenously (IV) and Azithromycin 500 mg once and 250 mg oral daily for five days were started for suspected community-acquired pneumonia. 

Three sets of sputum acid-fast bacilli (AFB) with mycobacterial culture showed negative results as well. Coccidioides complement fixation antibody test, *Bordetella pertussis* DNA polymerase chain reaction (PCR), *Chlamydia pneumoniae *DNA PCR, Histoplasma antigen, *Mycoplasma pneumoniae *PCR were all unrevealing. 

Bronchoscopy with bronchoalveolar lavage was performed. Bacterial culture along with AFB and fungal stain were negative. The AFB and fungal cultures were performed per protocol. Cytology was negative for malignancy, and Gomori methenamine silver stain (GMS) and periodic acid-Schiff (PAS) stain failed to demonstrate evidence of *Pneumocystis jirovecii* and fungi, respectively. Diagnosis of EVALI was reached and the patient was discharged with outpatient follow up with the pulmonologist. 

## Discussion

E-cigarettes usage or vaping is becoming more popular as an alternative option to cigarette smoking. Vaping is associated with a wide degree of pulmonary injuries such as asthma, chronic obstructive pulmonary disease or EVALI [5]. E-cigarette or vaping product use-associated lung injury is an acute or subacute respiratory illness that has become more recognized in recent years. However, it remains underdiagnosed [6]. It is a diagnosis of exclusion, and criteria commonly used to establish the diagnosis of EVALI include the use of an e-cigarette or related product such as vaping or dabbing in the previous 90 days, lung opacities on chest radiograph or CT, exclusion of lung infection, absence of a likely alternative diagnosis i.e., cardiac, neoplastic, rheumatologic [4,7]. The patient's respiratory illness was presumed to be EVALI after an extensive workup showed no clear cause of disease. The majority of patients with EVALI present with constitutional symptoms such as fever, chills or respiratory symptoms such as dyspnea, cough, pleuritic chest pain, and hemoptysis. However, some can present with gastrointestinal symptoms such as nausea, vomiting, diarrhea and abdominal pain [4]. 

The pathogenesis of EVALI is unknown. However, it appears to be a form of acute lung injury that represents a spectrum of disease processes, rather than a single disease process. The common chest radiographic finding associated with EVALI is diffuse hazy or features consolidative opacities [3]. Chest CT findings in EVALI most commonly show ground-glass opacities with subpleural sparing [8,9]. A miliary pattern of nodules on lung imaging is rarely associated with EVALI. It is more commonly seen with miliary tuberculosis (TB), which it is named after. The optimal treatment of EVALI is unknown, a trial of systemic corticosteroids has been given to the majority of patients but further data is needed to prove their efficacy [4,10,11]. The mainstay is the immediate cessation of e-cigarette or vaping use, followed by supportive care and supplemental oxygen.

Miliary TB on the other hand, is a form of tuberculosis that results from hematogenous dissemination of *Mycobacterium tuberculosis,* affecting multiple organs and systems. It can arise as a primary progressive infection and can occur as reactivation of latent TB. Clinical manifestations of miliary TB are most likely to be subacute or chronic, less likely to be acute and depend on the organ systems involved. Common manifestations are fever of unknown origin, failure to thrive, and night sweats [12-14]. The classic radiographic appearance of miliary TB is reticulonodular infiltrate and other less common features include pleural reactions, hilar or mediastinal adenopathy, interstitial or alveolar infiltrates, or cavities. A CT of the chest is more sensitive than chest radiography and involves multiple small nodules throughout the lung resembling millet seeds [15,16]. The approach to antimicrobial therapy for the treatment of miliary TB is the same as for pulmonary TB although drug-resistant strains may require modifications. Children, immunocompromised patients, patients with central nervous system involvement may require a longer duration of therapy [17].

## Conclusions

Common radiographic patterns associated with EVALI have been noted with features suggestive of diffuse alveolar hemorrhage, acute eosinophilic pneumonia, hypersensitivity pneumonitis, ​​organizing pneumonia or lipoid pneumonia. The presented case above describes new radiologic findings associated with EVALI. E-cigarette or vaping product use-associated lung injury is a newly described illness. This case will highlight and add to a growing body of literature regarding radiologic manifestations associated with EVALI. 
